# Expression of TIM-3 on Plasmacytoid Dendritic Cells as a Predictive Biomarker of Decline in HIV-1 RNA Level during ART

**DOI:** 10.3390/v10040154

**Published:** 2018-03-28

**Authors:** Albert Font-Haro, Vaclav Janovec, Tomas Hofman, Ladislav Machala, David Jilich, Zora Melkova, Jan Weber, Katerina Trejbalova, Ivan Hirsch

**Affiliations:** 1 Institute of Molecular Genetics of the Czech Academy of Sciences, 14220 Prague, Czech Republic; afont.haro@gmail.com (A.F.-H.); vaclav.janovec@natur.cuni.cz (V.J.); katerina.trejbalova@img.cas.cz (K.T.); 2 Department of Genetics and Microbiology, Charles University, Faculty of Sciences, BIOCEV, 25242 Vestec, Czech Republic; tomas.hofman@volny.cz; 3 Institute of Organic Chemistry and Biochemistry of the Czech Academy of Sciences, IOCB & Gilead Research Center, 16610 Prague, Czech Republic; weber@uochb.cas.cz; 4 The Third Faculty of Medicine, Charles University and Hospital Na Bulovce, 18081 Prague, Czech Republic; ladimachala@centrum.cz; 5 The First Faculty of Medicine, Charles University and Hospital Na Bulovce, 18081 Prague, Czech Republic; david.jilich@centrum.cz; 6 Department of Immunology and Microbiology, Charles University, The First Faculty of Medicine, BIOCEV, 25242 Vestec, Czech Republic; zmelk@lf1.cuni.cz

**Keywords:** HIV-1, antiretroviral therapy (ART), innate and adaptive immune responses, plasmacytoid dendritic cells (pDCs), pDC dysfunction, T cell Ig and mucin-domain containing molecule 3 (TIM-3), BDCA-2, Toll-like receptors 7 and 9 (TLR7/9)

## Abstract

Depletion and functional impairment of circulating plasmacytoid dendritic cells (pDCs) are characteristic attributes of HIV-1-infection. The mechanism of dysfunction of pDCs is unclear. Here, we studied the development of phenotype of pDCs in a cohort of HIV-1-infected individuals monitored before the initiation and during a 9-month follow up with antiretroviral therapy (ART). Using polychromatic flow cytometry, we detected significantly higher pDC-surface expression of the HIV-1 receptor CD4, regulatory receptor BDCA-2, Fcγ receptor CD32, pDC dysfunction marker TIM-3, and the marker of killer pDC, TRAIL, in treatment-naïve HIV-1-infected individuals before initiation of ART when compared to healthy donors. After 9 months of ART, all of these markers approached but did not reach the expression levels observed in healthy donors. We found that the rate of decline in HIV-1 RNA level over the first 3 months of ART negatively correlated with the expression of TIM-3 on pDCs. We conclude that immunogenic phenotype of pDCs is not significantly restored after sustained suppression of HIV-1 RNA level in ART-treated patients and that the level of the TIM-3 expressed on pDCs in treatment naïve patients could be a predictive marker of the rate of decline in the HIV-1 RNA level during ART.

## 1. Introduction

Plasmacytoid dendritic cells (pDCs) are a highly-specialized subset of dendritic cells that play a central role at the interface of innate and adaptive immunity. They sense HIV-1 primarily via endosomal Toll-like receptors 7 (TLR7), which recognizes ssRNA [1,2]. TLR signaling leads to the secretion of proinflammatory cytokines and chemokines such as interleukin 1 (IL-1), tumor necrosis factor α (TNF-α), IL-6, IL-8, and, most importantly, type I IFNs (IFN-I, α/β/ω) [3,4,5,6]. In addition to TLR7/9, pDCs express multiple specific receptors that facilitate antigen capture and presentation and regulate pDC function, namely IFN-I production, thus preventing abnormal immune response.

The role of IFN-I and pDCs in the pathogenesis of HIV-1 infection is unclear and ambivalent. IFN-I production is critical in the early phases of the immune response to infections, but the chronic and systemic activation of pDCs can paradoxically lead to deleterious consequences for the immune system [7,8]. It is likely that an intense chronic immune activation occurs in the mucosa, involving the accumulation of pDCs producing IFN-I during HIV-1 infection [9,10]. pDCs suppress HIV-1 replication but contribute to HIV-1 induced immunopathogenesis in humanized mice [11]. It has been shown that HIV-1 infection impairs B and T lymphocyte attenuator (BTLA)-mediated signaling in CD4^+^ and CD8^+^ cells dependent on pDC-derived IFN-α, which contributes to broad T-cell hyperactivation [12]. Chronic HIV-1 infection also depletes group 3 innate lymphoid cells (ILC3s) through pDC activation, induction of IFN-I, and CD95-mediated apoptosis, resulting in an increase in bacterial infection and inflammation [13].

In HIV-1 infection, the number of pDCs as well as their function is decreased simultaneously with CD4^+^ T cell population [14,15]. In contrast to immune activation, pDC dysregulation is directly related to the number of tolerogenic or apoptosis-inducing functions. Thus, upon infection, HIV-1 virions stimulate conversion of pDCs into TNF-related apoptosis-inducing ligand (TRAIL)-expressing IFN-producing killer pDC (IKpDC), which in turn facilitate the apoptosis of CD4^+^ T cells [16]. pDCs in untreated HIV-1-infected individuals compared to controls and ART-treated patients were also found to express an increased level of programme death ligands (PD-L) 1 and PD-L2 [17]. Another marker of pDC dysfunction in HIV-1-infected individuals, the T cell inhibitory receptor TIM-3 (T cell Ig and mucin-domain containing molecule) has been shown to be associated with the recruitment of IRF7 and p85 to lysosomes and the submembrane displacement of TLR9 [18].

To better understand the mechanisms underlying HIV-1 immunopathogenesis, we analyzed stimulatory, inhibitory, tolerogenic, or apoptosis-inducing functions that are mediated by pDCs in a cohort of HIV-1-infected individuals before and during a 9-month follow-up course of ART. In this cohort, we studied the expression of two HIV-1 receptors, CD4, a key determinant of divergent HIV-1 sensing by pDCs [19] and blood dendritic cell antigen 2 (BDCA-2, CD303, CLEC4C), a lectin-like regulatory receptor [20] which binds to and can be activated by the envelope glycoprotein gp120 of HIV-1 [21]. We also determined the expression of the pDC activation marker HLA-DR, Fcγ receptor CD32, pDC dysfunction marker TIM-3, and the killer pDC marker, TRAIL. We found that the frequency and the mean fluorescence intensity (MFI) of TIM-3^+^ pDCs determined before initiation or after 3 months of ART negatively correlated with the rate of decline in HIV-1 RNA level over the 3-month ART. Our data showed that the immunogenic phenotype of pDCs was not significantly restored after sustained suppression of HIV-1 RNA in 9-month ART-treated patients.

## 2. Materials and Methods

### 2.1. Ethics Statement

This study was conducted according to the principles expressed in the Declaration of Helsinki. Each patient provided informed written consent to participation in this study in accordance with institutional and regulatory guidelines. The study was approved by the Institutional Ethics Committee (Review Board) Na Bulovce Hospital in Prague, Czech Republic, registration number 22.3.2013/6637/EK-Z (22 March 2013).

### 2.2. Patients

Twenty-one viremic individuals with plasma viremia ≥10^4^ RNA copies per milliliter of plasma were enrolled for a period of 9 months at the HIV Clinic of Hospital Na Bulovce (Table 1) together with 16 sex-matched controls. The difference in age distribution of HIV-1-infected individuals (median (interquartile range (IQR))) 28, IQR (25–37) years and healthy controls 34, IQR (31–37) years was not statistically significant (*t*-test, *p* = 0.26). The first day of therapy and at the same time the first day of the blood sampling was determined individually for each patient according to recommended therapeutic criteria. Blood samples (10 mL) were collected before and after suppression of viral load by antiretroviral therapy, as shown in Table 1. We had access to the clinical data of these patients including analyses of their lymphocyte populations for another 14 months. Enrollment criteria: HIV-1 infection, ≥10^4^ HIV-1 viral copies/mL of plasma, treatment-naive state. Exclusion criteria: <18 years, HCV coinfection (patients must be HCV PCR negative). The efficiency of ART was determined using a COBAS AmpliPrep/COBAS TaqMan HIV-1 Test, version 2.0 (Roche, Basel, Switzerland).

### 2.3. Patients PBMCs

Patients’ PBMCs were separated using a BD Vacutainer CPT™ Cell Preparation Tube (BD Medical, Franklin Lakes, NJ, USA) according to the manufacturer’s instructions. Briefly, PBMCs were separated by density gradient centrifugation, then washed twice with PBS and used in the ensuing experiments.

### 2.4. In Vitro pDC Stimulation

To determine cytokine production, PBMCs aliquoted in 100-µL quantities (10^7^ cells/mL) into 96-well round-bottom culture plates and stimulated with 20 µg/mL of BDCA-2 mAb or IgG1 isotype for 2 h and then with 4 µg/mL of CpG-A for 16 h.

### 2.5. Flow Cytometry Analysis

To carry out the flow cytometric analysis of pDC phenotype, we created a muticolor panel composed of PerCP/Cy5.5-CD11c, BV421-BDCA2, APC-TRAIL, PE-CD4, APC-Fire750-TIM3, FITC-Lin1 (all from Biolegend, San Diego, CA, USA), and V500-HLADR, BV605-CD32 (from BD Biosciences, San Jose, CA, USA). The staining was performed in Brilliant Stain Buffer (BD Biosciences) as recommended by the manufacturer. Cells were fixed in 4% paraformaldehyde and data were acquired within 48 h. We included Lin1-FITC-labeled antibody along with Zombie Green fixable viability dye (Biolegend, San Diego, CA, USA) in a dump channel. We used an LSR Fortessa SORP (Becton Dickinson, San Jose, CA, USA) cytometer equipped with 5 non-colinear lasers and 20 detectors. A final analysis of flow cytometry data was carried out using FlowJo software (Tree Star, Inc., Ashland, OR, USA). Routine analyses of the major lymphocyte populations (FITC-A-CD3, PerCP-Cy5.5-CD45, PE-Cy7-CD4, APC-Cy7-CD8, APC-CD19, PE-CD16+56) in peripheral blood of ART-treated HIV-1-infected individuals were performed using a BD FACSCanto II flow cytometer (Becton Dickinson).

### 2.6. Statistical Analysis

Quantitative variables were expressed as the mean ± SEM (standard error of the mean). To compare the levels of cytokine production by pDCs, we used either Mann–Whitney or a Wilcoxon two-tailed non-parametric tests. The data was analyzed with GraphPad Prism 4 (GraphPad Software, La Jolla, CA, USA). A *p* value of ≤0.05 was considered to be significant.

## 3. Results

### 3.1. Persistent Dysfunction of pDCs from ART-Treated HIV-1-Infected Individuals after Sustained Suppression of HIV-1 RNA

**Figure 1:**
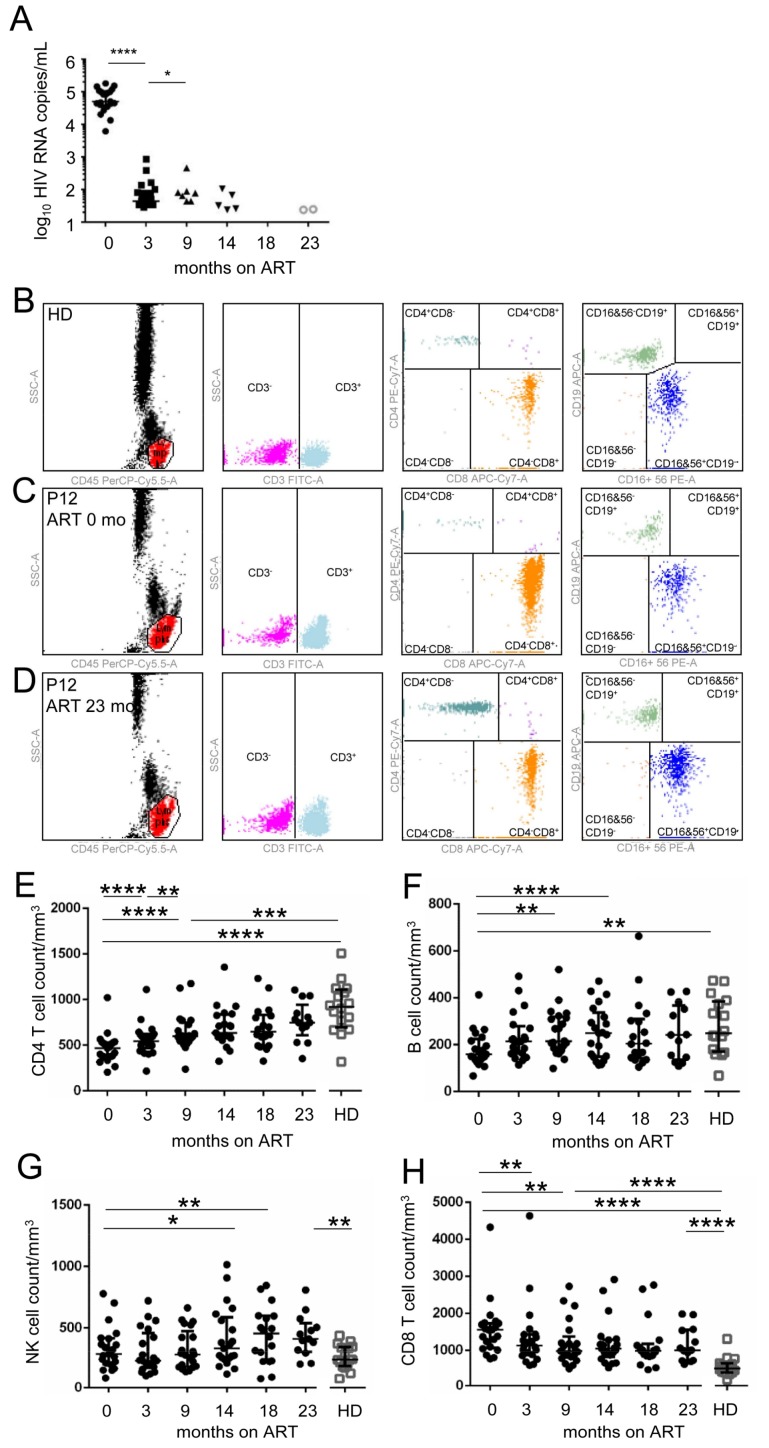
Adaptive immunity is only partially restored over the course of ART despite the sustained suppression of HIV-1 RNA level. Characteristics of the cohort of 21 HIV-infected subjects. (**A**) HIV RNA copies/mL of plasma before and during anti-retrovirus therapy (ART). (**B**–**D**) Dot plots for the quantification of the major lymphocyte populations in peripheral blood of a healthy donor (HD) (**B**), treatment-naïve patient no. 12 (P12) (**C**), the same patient after 23 months of ART (**D**). (**E**) CD3^+^CD4^+^ T cell counts during ART. (**F**) CD19^+^ B cell counts during ART. (**G**) CD3^−^CD16^+^CD56^+^ NK cell counts during ART. (**H**) CD3^+^CD8^+^ T cell counts during ART. The data show medians and interquartile range, N = 21. * *p* < 0.05; ** *p* < 0.01; *** *p* < 0.001; **** *p* < 0.0001; two-tailed paired Wilcoxon test.

Here, we analyzed the main types of immune cells in peripheral blood in a cohort of 21 ART-treated HIV-1-infected individuals (Table 1). We assessed the dynamics of the lymphocyte populations for a period of 23 months (Figure 1). After 3 months of ART, we observed a decrease in plasma HIV-1 RNA level from (median (interquartile range (IQR))) 4.70, IQR (4.57–5.02) log_10_ copies/mL 1.64, IQR (0.7–1.96) log_10_ copies/mL, below the level of 2.93 log_10_ copies/mL of plasma, and it continued to decrease over the remaining 6 months (Figure 1A). The major lymphocyte populations, CD4^+^ and CD8^+^ T cells, B cells, and NK cells in healthy donors, treatment naïve HIV-1-infected patients, and ART-treated HIV-1-infected individuals were quantified by flow cytometry (Figure 1B–D). Inversely to HIV-1 RNA level, CD4^+^ T cell count in blood increased from the median value of 469, IQR (375–531) CD4^+^ T cells/mm^3^ of blood to 748, IQR (609–945) CD4^+^ T cells/mm^3^ of blood (Figure 1E). Similarly, the median value of B cells in blood increased from 159, IQR (137–224) B cells/mm^3^ of blood to 214, IQR (136–367) B cells/mm^3^ of blood (Figure 1F). Also, the median value of NK cells increased from 230, IQR (169–459) NK cells/mm^3^ of blood to 412, IQR (308–541) NK cells/mm^3^ of blood (Figure 1G). In contrast to CD4^+^ T cells, B cells, and NK cells, the median value of CD8^+^ T cells decreased from 1551, IQR (1070–1737) CD8 T cells/mm^3^ of blood to 1005, IQR (713–1555) CD8 T cells/mm^3^ of blood (Figure 1H). After 9 months of ART, the median values of CD8^+^ T cells remained significantly higher than those in healthy donors (HD), while the median values of CD4^+^ T cells remained significantly lower. The CD8^+^ T cell levels were significantly increased in comparison to healthy donors even after 23 months of ART.

**Figure 2:**
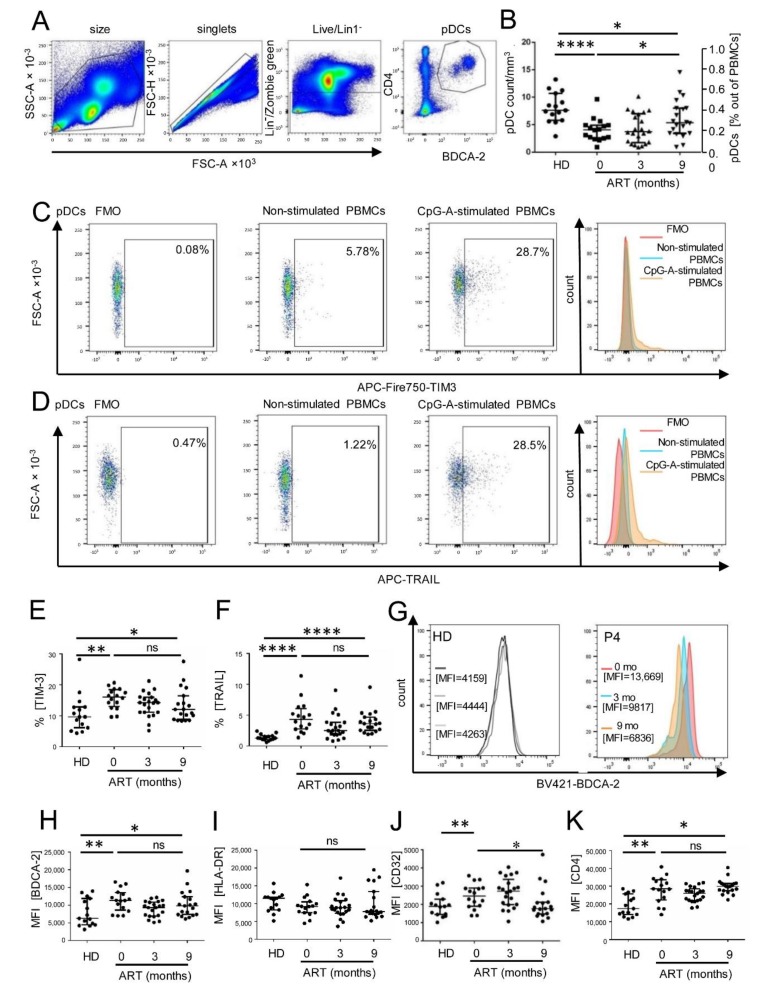
Impaired pDC phenotype persists after sustained suppression of HIV RNA in plasma of ART-treated patients. (**A**) Gating strategy for identification of pDC phenotype: PBMCs were gated according to their size and then into singlets, and after exclusion of dead cells (Zombie green) and Lin1^+^ cells into a CD4^+^BDCA-2^+^ pDC population. (**B**) pDC number and proportion of PBMCs in healthy donors (HD) and in the course of ART. (**C**,**D**) Dot plots and histograms for the quantification of TIM3 (**C**) and TRAIL (**D**) in Lin^−^CD4^+^BDCA-2^+^ live pDCs are shown. FMO was used for gating TIM-3^+^ (**C**) and TRAIL^+^ (**D**) pDCs in mock-stimulated or CpG-A-stimulated PBMCs from a healthy donor and from HIV-1-infected patients (**E**,**F**). (**E**) The frequency of pDCs expressing TIM-3 in the cohort of 21 patients (**F**) The frequency of pDCs expressing TRAIL. (**G**) Examples of histograms for the quantification of BDCA-2 in three healthy donors (HD) and ART-treated patient no. 4 (P4) determined 0, 3, and 9 months after therapy initiation. (**H**) The MFI of BDCA-2^+^ expressed on pDCs. (**I**) The MFI of HLA-DR^+^ expressed on pDCs. (**J**) The MFI of CD32^+^ expressed on pDCs. (**K**) The MFI of CD4^+^ expressed on pDCs. The data show medians and interquartile ranges. N = 21. * *p* < 0.05; ** *p* < 0.01; **** *p* < 0.0001; ns, non-significant; two-tailed Mann-Whitney test.

While the dysfunction of CD4^+^, CD8^+^ T cells, B cells, and NK cells during the course of HIV infection has been extensively studied, little is known regarding the impairment of dendritic cell function. According to design of this study, we followed the development of pDC cell count and phenotype during 9 months of ART. Previous reports showed that the quantity of blood pDCs is severely reduced in AIDS patients [14,15]. To quantify pDCs, PBMCs were gated according to their size and then into singlets, and after exclusion of dead cells, pDCs were defined as live Lin^−^CD4^+^BDCA2^+^ cells (Figure 1A,B). The pDC median number in the cohort of 21 treatment-naïve HIV-1-infected individuals before initiation of ART was 4.08, IQR (2.59–4.90) pDC/mm^3^. It was reduced by 54% in comparison to 13 healthy donors (Figure 2B). Nine months of ART partially restored pDC cell count but its median value 5.35, IQR (3.45–7.99) pDC/mm^3^ remained significantly lower than that detected in healthy donors (71%, *p* = 0.04). Increase of the median cell number in pDCs (1.31 times, *p* = 0.04, Figure 2A) observed over 9 months of ART was like that observed for CD4^+^ T cells (1.28 times, *p* = 0.008, Figure 1B).

Then, we investigated the expression of surface markers TIM-3, TRAIL, BDCA-2, HLA-DR, CD32, and CD4 in Lin^−^CD4^+^BDCA-2^+^ pDCs (Figure 2A,C–K). FMO gating was used to quantify the frequency of TIM-3^+^ and TRAIL^+^ pDCs, while the mean fluorescence intensities (MFI) were used to quantify expression of constitutive pDC markers, BDCA-2, HLA-DR, CD32, and CD4. As reported previously, HIV-1 infection in treatment-naïve individuals is associated with elevated frequency of TIM-3^+^ pDCs [18]. We found that the frequency of TIM-3^+^ pDCs in treatment-naïve individuals exceeded 1.42 times the frequency of TIM-3^+^ pDCs in healthy donors (*p* = 0.0026, Figure 2E). The median value of the frequency of TIM-3^+^ pDCs showed decreasing tendency over the 9-month ART but their median value remained significantly elevated in comparison to healthy donors (1.2 times, *p* = 0.0155). Similarly, HIV-1 infection in treatment-naïve individuals was associated with elevated median value of the frequency of TRAIL^+^ pDCs (3.32 times, *p* < 0.0001), which showed decreasing tendency over the 9-month ART (Figure 2F). The frequency of TRAIL^+^ pDCs in ART-treated patients remained significantly elevated in comparison to healthy donors (2.76 times, *p* < 0.0001).

Then, we analyzed expression of the pDC markers that are constitutively present in pDCs. As shown for BDCA-2, we compared the MFI values of these markers in healthy donors with those of ART-treated HIV-1-infected individuals (Figure 2G). In comparison to healthy donors, HIV-1 infection in treatment-naïve individuals was associated with elevated MFI of BDCA-2 (1.8 times, *p* = 0.015) (Figure 2H), CD32 (1.5 times, *p* = 0.046) (Figure 2J) and CD4 (1.6 times, *p* = 0.0013) (Figure 2K). Among all these variables, only the median values of MFIs of HLA-DR did not significantly vary between healthy donors, treatment-naïve, and ART-treated HIV-1-infected individuals (Figure 2I). The median values of MFIs of these markers showed a decreasing tendency in the course of ART, however, with the exception of CD32, which decreased 1.38 times (*p* = 0.044), they did not reach statistical significance; median values of CD4 and BDCA-2 of ART-treated patients remained significantly elevated in comparison to healthy donors.

Collectively, based on this multifactorial analysis we concluded that after sustained suppression of the HIV RNA level in 9-month ART-treated patients, the median number of pDCs significantly increased, however, their immunogenic phenotype was not significantly restored.

### 3.2. Decline in HIV-1 RNA Level after Initiation of ART Correlates with Expression of TIM-3 on pDCs

**Figure 3:**
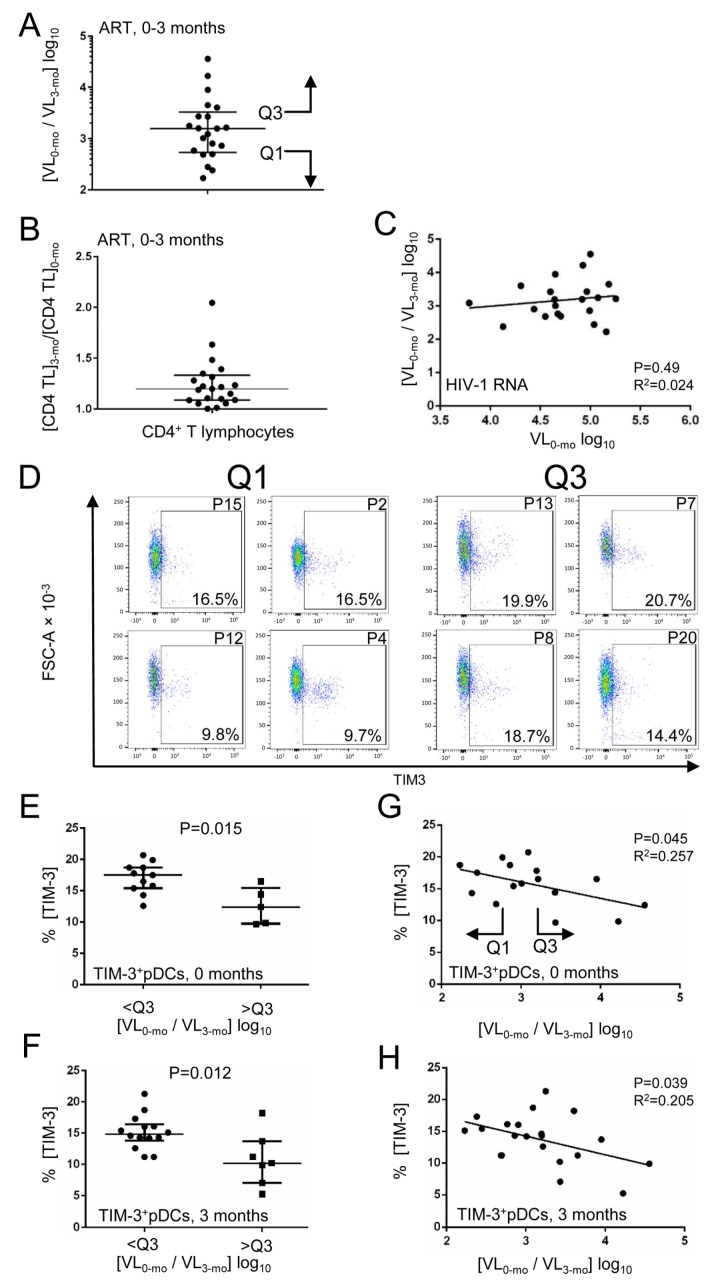
Expression of TIM-3 on pDCs of HIV-1-infected individuals negatively correlates with the rate of decline in HIV-1 RNA copies/mL of plasma over the 3-month ART. (**A**) The rate of decline in HIV-1 virus load (VL) expressed as [VL_0-mo_/VL_3-mo_] log_10_, where VL_0-mo_ is HIV-1 RNA copy number/mL in treatment-naïve individuals (zero time of ART) and VL_3-mo_ is HIV-1 RNA copy number/mL after 3 months of ART in the cohort of 21 HIV-1-infected individuals. (**B**) The recovery rate of CD4^+^ T cells in HIV-1-infected individuals expressed as a ratio of CD4 T cell number after 3 months of ART [CD4 TL]_3-mo_ to CD4 T cell number at zero time of ART [CD4 TL]_0-mo_. (**C**) The rate of decline in HIV-1 RNA copies/mL does not correlate with HIV-1 VL in treatment-naïve individuals (zero time of ART). (**D**) Examples of dot plots for the quantification of TIM3 in Lin^−^BDCA-2^+^-gated live pDCs are shown for PBMCs from treatment-naïve individuals from Q1 (patients P2, P4, P12, P15) and Q3 (patients P7, P8, P13, P20). (**E**,**F**) Comparison of the frequency of TIM-3^+^ pDCs in treatment-naïve HIV-1-infected patients (**E**) or patients after the 3-month ART (**F**), in which [VL_0-mo_/VL_3-mo_] log_10_ was <Q3 or >Q3. (**G**,**H**) Correlation of the frequency of TIM-3 expressed on pDCs in treatment-naïve HIV-1-infected patients (**G**) or patients after the 3-month ART (**H**) with [VL_0-mo_/VL_3-mo_] log_10_ (the same samples as in panels (**E**,**F**) were analyzed). The data show medians and interquartile ranges. Q1, the first quartile; Q3, the third quartile; *p* < 0.05 was considered to be significant; two-tailed Mann–Whitney test.

We defined the rate of decline in HIV-1 RNA level over the 3-month ART as a new additional parameter to evaluate the success rate of ART in HIV-1-infected individuals. To this end we calculated the ratio of plasma HIV-1 RNA copies/mL (virus load, VL) determined in the treatment-naïve individuals at the time zero of ART (VL_0-mo_) to the plasma HIV-1 RNA copies/mL over the 3-month ART (VL_3-mo_) (Figure 3A, Table 1). While the HIV-1 RNA level in different patients decreased over the first 3 months of ART by 2.2–4.6 log_10_ (Figure 3A), CD4^+^ T cell count increased over the same period of time from 1.1 to 2.8 times (Figure 3B). The rate of decline in HIV-1 RNA level over 3 months did not correlate with the initial virus load in the same individuals (Figure 3C), or with the rate of restoration of CD4^+^ T cells. We used the rate of decline in plasma HIV-1 RNA copies/mL over 3 months [VL_0-mo_/VL_3-mo_] log_10_ as a parameter to characterize individuals that respond more or less rapidly to ART. We subsequently analyzed the distribution of phenotypic markers of pDCs in these HIV-1-infected individuals and addressed the question of whether this approach can define bona fide groups of slow and rapid responders to ART. First, using the third quartile Q3 of the decline rate as a parameter, we found that the frequency of TIM-3^+^ pDCs detected in HIV-1-infected individuals before ART (Figure 3D,E) and in the same patients over the 3-month ART (Figure 3F) was significantly higher in slowly responding ([VL_0-mo_/VL_3-mo_] log_10_ < Q3) than in rapidly responding ([VL_0-mo_/VL_3-mo_] log_10_ > Q3) individuals (*p* = 0.015 before ART, *p* = 0.012 after 3-month ART). TIM-3 was the only phenotypic marker analyzed in this study that correlated with the rate of decline in plasma HIV-1 RNA copies/mL. Then, we analyzed correlation of the frequency of TIM-3^+^ pDCs in the whole cohort of 21 HIV-1-infected individuals with the rate of decline in plasma HIV-1 RNA (Figure 3G,H). In both HIV-1-infected individuals before ART (Figure 3G) and in the same patients over the 3-month ART (Figure 3H), the rate of decline in HIV-1 RNA level significantly correlated with the frequency of TIM-3^+^ pDCs. In contrast, correlation of the total HIV-1 RNA level (VL_0-mo_ log_10_ copies/mL) before ART with the frequency of TIM-3^+^ pDCs was not significant (*p* = 0.44).

## 4. Discussion

Although multiple markers of active immune state are significantly reduced by ART, immune activation following sustained suppression of HIV-1 RNA in plasma remains significantly elevated when compared to uninfected controls [22,23,24,25,26]. Using a cohort of patients who were subjected to multiple samplings before and 3 and 9 months after ART initiation, we demonstrated partial restoration of adaptive immune function, as evidenced by the increase in the average number of CD4^+^ T cells and B cells to standard levels of healthy donors [27,28,29]. In contrast, the average numbers of NK cells (CD3^−^/CD16^+^/CD56^+^) and CD8^+^ T cells remained significantly higher over the standard reference ranges in healthy individuals [27,28,29].

Our results show that over a period of 9-months, ART partially restored pDC numbers, however, the immunogenic phenotype of pDCs was not significantly restored. While impairment of single pDC functions was demonstrated in several reports [15,18,19,30,31], in the present study we analyzed a complex dysfunction of pDC covering expression of the MHC class II ligand, the high affinity HIV-1 receptor CD4, the regulatory receptor BDCA-2, the Fcγ receptor CD32, the pDC dysfunction marker TIM-3, and the marker of killer pDC, TRAIL.

We found that TIM-3 was the only phenotypic markers among the pDC markers analyzed in this study whose expression correlated with the rate of decline in HIV-1 RNA level after the initiation of ART. Correlation of the rate of decline in HIV-1 RNA copies with the TIM-3 expression level at both time 0 and over 3 months of ART is consistent with sustained expression of TIM-3 during ART. We suggest that the rate of decline in HIV-1 RNA level during the first period after initiation of ART, in our case 3 months, could be a new additional parameter to characterize response to ART. A higher number of enrolled patients and a longer follow-up period will be necessary to evaluate the possible clinical significance of this parameter.

Several immune mechanisms participate in clearance of HIV-1 during ART. Among them, impaired production of IFN-α and TNF-α regulated in pDCs by TIM-3 can play an important role in the immunopathogenesis of HIV-1 infection [18]. During HIV-1 infection, pDC-activating TLR7/9 agonists induce TIM-3 expression and subsequently result in the impairment of pDC function. It was shown that IFN-α and TNF-α production was impaired in TIM-3^+^ pDCs, and that TIM-3 may transfer TLR agonists into acidic lysosomes, bypassing TLR activation [18]. The high level of colocalization of TIM-3 and IRF7 within lysosomes also suggests a mechanism by which TIM-3 may regulate IFN-α production. 

The molecular mechanism of depletion of circulating pDCs accompanied by the impaired secretion of IFN-I and proinflammatory cytokines and capacity of antigen presentation remains elusive despite years of intense research [4,5,6,20,32,33,34,35]. HIV-1-exposed pDCs express an increased level of markers of pDC dysfunction, as PD-L1, PD-L2, TIM-3, and BDCA-2. The increased level of the median value of MFIs of BDCA-2 in pDCs of HIV-1-infected individuals in comparison to healthy donors is of special interest. In contrast to MFI, the frequency of BDCA-2^+^ pDCs is not influenced by HIV-1 infection, and in vitro activation of pDC via TLR7/9 agonists leads to downregulation of the pDC surface-localized BDCA-2 [14]. Thus, some other signals should be responsible for increased expression level of BCDA-2 on pDCs of HIV-1-infected individuals. Signaling via pDC regulatory receptors, including BDCA-2, attenuates TLR-induced production of IFN-I and proinflammatory cytokines [4,5,6,20,33,35,36,37]. Although by different mechanisms, TIM-3 also inhibits production of IFN-α and TNF-α in pDCs. IFN control is hijacked in the pathogenesis of several chronic viral infections including HIV-1, leading to immune tolerance [6,21,38,39].

## 5. Conclusions

We conclude that the immunogenic phenotype of pDCs is only partially restored after sustained suppression of HIV RNA level in ART-treated patients and that a high level of pDC-expressed TIM-3 in treatment naïve patients could be a useful predictive biomarker of a slow decline in HIV-1 RNA level during ART. Establishing the clinical significance and generalizability of this observation will require larger numbers of patients and more extended follow-ups with clinical outcomes.

## Figures and Tables

**Table 1:** Clinical characteristics of the HIV patient cohort.

Subject No. ^1^	Transmission ^2^	Age	Diagnosis-Initiation of ART (Months)	Therapy Regimen ^3^	CD4^+^ T Cells (Cell/mm^3^) 0-Month ART	CD4^+^ T Cells (Cell/mm^3^) 3-Month ART	HIV-1 RNA (Copies/mL) VL_0-month_^4^	HIV-1 RNA (Copies/mL) VL_3-month_^5^
1	MSM	26	4	ABC/3TC+RPV	336	498	44,800	44
2	MSM	23	3	ABC/3TC+EFV	468	599	44,600	<20
3	MSM	25	52	ABC/3TC+DRV/r	565	676	50,600	102
4	MSM	22	10	TDF/FTC+EFV	514	624	92,100	34
5	MSM	27	10	ABC/3TC+DRV/r	527	644	153,000	34
6	Bi	44	8	ABC/3TC+EFV	480	530	82,700	52
7	MSM	26	12	TDF/FTC/RPV	1023	1111	6150	<20
8	MSM	27	5	TDF/FTC+DRV/r	315	438	144,000	850
9	MSM	38	15	TDF/FTC/EVG/c	521	598	27,300	34
10	Bi	49	62	ABC/3TC+DRV/r	379	619	119,000	67
11	Bi	48	6	ABC/3TC+DRV/r	372	501	20,200	<0
12	MSM	29	4	ABC/3TC+LPV/r	267	546	83,900	<20
13	MSM	31	1	TDF/FTC/RPV	402	528	47,300	81
14	MSM	37	13	TDF/FTC/EVG/c	634	782	99,700	61
15	MSM	36	11	TDF/FTC+DRV/r	503	597	180,000	<20
16	MSM	28	32	TDF/FTC+DRV/r	206	217	98,100	135
17	MSM	24	14	TDF/FTC/RPV	377	414	35,400	73
18	MSM	19	4	ABC/3TC+DRV/r	418	418	109,000	391
19	MSM	26	21	TDF/FTC/RPV	534	540	43,900	28
20	MSM	44	4	TDF/FTC+DTG	538	585	39,900	166
21	MSM	28	25	TDF/FTC+DTG	384	404	13,400	<20

^1^ All subjects were males; ^2^ MSM (men who has sex with men), Bi (bisexual); ^3^ ABC (Abacavir); 3TC (lamivudine); TDF (tenofovir); FTC (emtricitabine); RPV ; EFV (efavirenz); DRV (darunavir); EVG (elvitegravir); LPV (lopinavir); DTG (dolutegravir); r (ritonavir) and c (cobicistat) are pharmacokinetic enhancers; ^4^ HIV-1 virus load (plasma HIV-1 RNA (copies/mL)) at time zero of ART; ^5^ HIV-1 virus load (plasma HIV-1 RNA (copies/mL)) 3 months after initiation of ART.
